# Assessment of short- and long-term outcomes of aortic valve-sparing operation at concomitant aortic root and arch repair

**DOI:** 10.1093/icvts/ivaf045

**Published:** 2025-02-26

**Authors:** Yu Hohri, Megan M Chung, Kavya Rajesh, Dov Levine, Christopher He, Elizabeth L Norton, Bradley Leshnower, Yanling Zhao, Paul Kurlansky, Edward P Chen, Hiroo Takayama

**Affiliations:** Division of Cardiothoracic and Vascular Surgery, New York Presbyterian Hospital/Columbia University Irving Medical Center, New York, NY, USA; Division of Cardiothoracic and Vascular Surgery, New York Presbyterian Hospital/Columbia University Irving Medical Center, New York, NY, USA; Division of Cardiothoracic and Vascular Surgery, New York Presbyterian Hospital/Columbia University Irving Medical Center, New York, NY, USA; Division of Cardiothoracic and Vascular Surgery, New York Presbyterian Hospital/Columbia University Irving Medical Center, New York, NY, USA; Division of Cardiothoracic Surgery, Emory University School of Medicine, Atlanta, GA, USA; Division of Cardiothoracic Surgery, Emory University School of Medicine, Atlanta, GA, USA; Division of Cardiothoracic Surgery, Emory University School of Medicine, Atlanta, GA, USA; Division of Cardiothoracic and Vascular Surgery, New York Presbyterian Hospital/Columbia University Irving Medical Center, New York, NY, USA; Division of Cardiothoracic and Vascular Surgery, New York Presbyterian Hospital/Columbia University Irving Medical Center, New York, NY, USA; Division of Cardiothoracic Surgery, Duke University Medical Center, Durham, NC, USA; Division of Cardiothoracic and Vascular Surgery, New York Presbyterian Hospital/Columbia University Irving Medical Center, New York, NY, USA

**Keywords:** valve-sparing aortic root replacement, aortic root surgery, aortic arch surgery, total arch replacement

## Abstract

**OBJECTIVES:**

Concomitant aortic root and arch replacement is a complex procedure. Although valve-sparing root replacement may offer advantages over valve prostheses, the decision to spare the valve may increase the risk profile of this procedure. This study examines the safety of aortic valve-sparing operation in such settings.

**METHODS:**

All patients who underwent concomitant aortic root and arch replacement between 2004 and 2021 at two aortic centres were reviewed. Patients with aortic stenosis, endocarditis or a history of previous cardiac surgery were excluded. Inverse probability treatment weighting yielded well-balanced cohorts. The primary end-points were mortality and complications during the index hospital stay, and secondary end-points were long-term survival and aortic valve reintervention rate.

**RESULTS:**

A total of 764 patients who underwent concomitant aortic root and arch replacement, including valve-sparing root replacement (*n* = 311) or composite valve graft root replacement (*n* = 453), were analysed. Surgical indication was dissection in 155 (20.2%), and distal extension was total arch replacement in 50 (6.5%). Cardiopulmonary bypass and cross-clamp times were longer in valve-sparing root replacement (*P* = 0.006 and *P* < 0.001, respectively). Valve-sparing root replacement demonstrated comparable in-hospital mortality rates (2.5% vs 4.9%, *P* = 0.195) and showed higher long-term survival rates (*P* = 0.04) (12-year survival rate; 78.5% [71.7–86.1%] vs 64.2% [57.4–71.6%]), which was reconfirmed on multivariable Cox regression analysis (hazard ratio: 0.505 [0.348–0.734], *P* < 0.001). The cumulative incidence of reintervention was similar in both groups (*P* = 0.62).

**CONCLUSIONS:**

In appropriately selected patients requiring aortic root and arch replacement, a valve-sparing operation may be performed safely without increased operative risk.

## GRAPHICAL ABSTRACT

**Figure:**
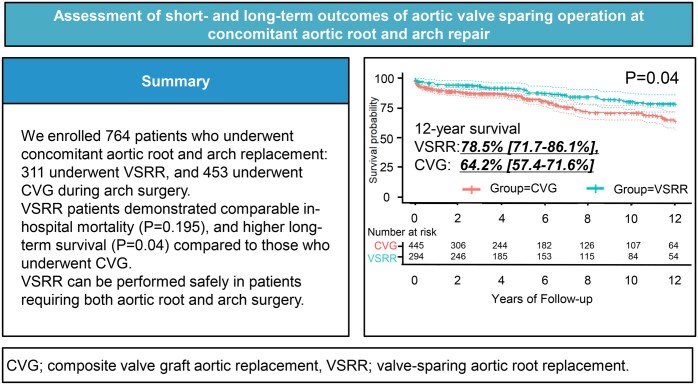

## INTRODUCTION

Valve-sparing aortic root replacement (VSRR) has been increasingly recognized as a useful and beneficial procedure and has become a standard surgical technique [1]. While this operation adds procedural complexity and time compared to replacing the aortic valve—as in a conventional composite valve graft root replacement (CVG)—its indications are expanding beyond aortic root aneurysm to include more complex pathologies, such as aortic dissection and bicuspid aortic valve (BAV) [1–3]. Though the conceptual benefits of this procedure may be understood, sparing the aortic valve during concomitant root and arch repair poses a unique challenge to surgeons, who must balance procedural risks against future benefits [4]. Several previous studies, including a meta-analysis, have compared 10- to 20-year outcomes between VSRR and CVG in patients with both aortic root aneurysm and dissection [5–12]. Although these studies include cases with concomitant arch surgery, there is a lack of literature specifically analysing outcomes after VSRR and CVG during arch surgery. These include some of the most complex and high-risk cases in aortic surgery—situations where the perioperative risk of sparing the valve may be prohibitively high in relation to the long-term benefits. To address this knowledge gap, we examined the safety of a valve-sparing operation at the time of concomitant root and arch repair by comparing outcomes of patients who have undergone combined aortic root and arch procedures with VSRR or CVG.

## PATIENTS AND METHODS

### Ethical statement

The study was approved by the Institutional Review Boards of Columbia University Irving Medical Center and Emory University with the waiver of consent (Columbia: AAAU0575, 4 April 2022; Emory: IRB00001479, 30 August 2021). Collection and storage of data from research participants for multiple and indefinite use strictly comply with requirements outlined in the WMA Declaration of Taipei. The IRB approved the establishment and continuously monitors ongoing use of the database.

### Study design and patient selection

This retrospective multicentre study included all patients undergoing aortic root replacement (ARR) between February 2004 and February 2021 at our centres. Inclusion criteria were patients who underwent ARR with concomitant arch surgery, specially hemiarch, partial arch (PAR) or total arch replacement (TAR). Hemiarch replacement is defined as the replacement of the ascending aorta with an open distal anastomosis. TAR involves replacing the entire aortic arch, from the innominate artery to the left subclavian artery, using any method of reimplantation or revascularization of the supra-aortic branches. PAR refers to cases where the distal anastomosis is proximalized into zone 2 [13]. Exclusion criteria were infrequent types of ARR procedures using allo- or autograft, as well as factors that clearly precluded valve-sparing operation, such as aortic stenosis and infective endocarditis. Reoperative cases were also excluded to reduce patient selection bias.

Baseline characteristics, operative details and in-hospital complications were collected through electronic medical records. Shared variables using the Society of Thoracic Surgeons (STS) Adult Cardiac Database definitions were used to create a merged database between the two aortic centres. The most recent clinical follow-up data were obtained between 2019 and 2022 through protocoled aortic centre visits or chart review and callbacks of patients and cardiologists. Survival status data were supplemented by the Centers for Disease Control and Prevention’s National Death Index (accessed 29 March 2022; data complete through 31 December 2021).

### Study end-point

The primary end-points were the short-term outcomes of in-hospital mortality and uneventful recovery. Uneventful recovery is a binary composite end-point describing any patient discharged from the hospital without in-hospital mortality or any of the four most common complications tracked in the STS database: stroke, reoperation for bleeding, prolonged ventilation or acute renal failure [14]. The secondary end-points were long-term survival and cumulative incidence of aortic valve reintervention.

### Aortic root and arch procedure

Surgical indications generally followed guidelines at the time [1]. For ARR, surgical types were determined taking several factors into consideration, including comorbidities; extent of necessary concomitant procedures; valve integrity issues with focal calcifications, fibrosis or fenestrations; and patient and surgeon preference. Neither severe AI, acute type A aortic dissection nor large aneurysm was an absolute contraindication for VSRR. The details of the VSRR procedure (David procedure) at each institution have been published previously [2, 9, 10]. When aortic valve replacement was required, the choice of prosthetic valve followed with the guidelines and patient’s preference [1]. Right axillary artery or distal ascending aorta was generally used for arterial cannulation site [15]. For arch replacement, the distal aortic anastomosis was performed typically with moderate hypothermia at a nasopharyngeal temperature range of 24°C to 28°C. The cerebral perfusion strategy depended on the complexity and extent of arch replacement and surgeon preference. In the majority of cases, antegrade cerebral perfusion was used as the cerebral perfusion strategy [16, 17]. When the right axillary artery was used for arterial cannulation, we continued perfusion through this cannulation. However, when the distal ascending aorta was used for arterial cannulation, we removed it and then inserted antegrade cerebral perfusion cannulas directly into the innominate artery, and if indicated into the left carotid artery. Distal systemic perfusion was temporarily halted until the distal aortic anastomosis was completed, after which systemic perfusion was immediately resumed. Near-infrared spectroscopy was utilized for the monitoring of regional oxygen saturation in the frontal lobes, and nasopharyngeal temperature was measured during surgery. For TAR, the supra-aortic vessels were individually reconstructed using a multi-branch graft. The sequence of distal and supra-aortic reconstruction was determined by the extent of arch replacement and surgeon preference. If the left subclavian artery was deep, distal anastomosis was proximalized into zone 2 [17, 18]. The details of the root and aortic arch at each institution have been previously described [2, 9, 10, 15–20].

### Statistical analysis

Continuous variables were expressed as mean (standard deviation) or median (interquartile range [IQR]) depending on normality of distribution, which was tested via the Shapiro–Wilk test, and were compared using Student’s *t*-test or the Mann–Whitney *U*-test, respectively as appropriate. Categorical variables were expressed as numbers and percentages and were compared using x^2^ test or Fisher’s exact test when appropriate. Missing data, which comprised a small proportion (<7.3%), were imputed using the Random Forest algorithm (Supplementary Table S1). Propensity score (PS)-based inverse probability of treatment weighting (IPTW) technique was used to construct balanced groups of patients who underwent either VSRR or CVG. Covariates included in the logistic regression propensity model were based on patient status at the time of the surgical decision and factors influencing the choice between types of ARR. These included age; sex; body mass index; diabetes; dyslipidaemia; chronic kidney disease; hypertension; cerebrovascular disease; peripheral artery disease; connective disorder disease; left ventricular ejection fraction; aortic insufficiency (AI); BAV; acute type A aortic dissection (AAAD); elective status; concomitant procedures such as hemiarch replacement, TAR/PAR, coronary artery bypass grafting and mitral surgery; and operation era. To reduce the impact of variations in clinical management and follow-up duration due to changes in the proportions of VSRR or CVG over different eras, we incorporated the operation era into the matching algorithm. The 18-year study period was divided into three equal intervals of six years: 2004–2009, 2010–2015 and 2016–2021. Patients were weighted by 1/PS for receiving VSRR and by 1/(1 − PS) for receiving CVG, and trimming was applied at the 2.5th and 97.5th percentiles of the PS distribution. Standardized mean differences (SMDs) were calculated to assess the balance between the two groups after applying the IPTW technique. Optimal balance was confirmed via SMD < 0.1. Overlap analysis was used to verify the distribution of patients included in the analysis (Supplementary Fig. S1). Long-term mortality was assessed using Kaplan–Meier methods and compared using the log-rank test. Multivariable cox regression identified the quantitative relationship between the type of ARR and patient outcomes. To minimize the variation in surgical management across institutions, ‘institution’ was included as a random effect. Variables were included in the regression model based on univariable analyses or clinical relevance; results from univariable regression are shown in Supplementary Table S2. No variables were found to be highly correlated using the variance inflation factor (all variance inflation factor < 5). Proportional hazards were confirmed via the Schoenfeld residuals. Cumulative incidence of aortic valve reintervention was graphed and compared via the Gray test. Several sensitivity analyses were conducted to test the robustness of our finding about long-term survival. First, E-value was calculated [21]. Second, considering the preferential influence of lower short-term mortality and morbidities in VSRR on long-term survival, we compared long-term survival in patients with uneventful recovery. Furthermore, to compare outcomes while minimizing heterogeneity in the complexity of aortic arch surgery, we conducted a subgroup analysis to evaluate the short- and long-term outcomes between VSRR and CVG with concomitant hemiarch replacement. A *P*-value of 0.05 was deemed significant for all analyses. All statistical analyses were performed using R version 4.3.0 (R Foundation for Statistical Computing, Vienna, Austria).

## RESULTS

### Patient characteristics and operative details: all patients

A total of 764 patients were included in the present study (VSRR: *n* = 311, CVG: *n* = 453) (Supplementary Fig. S2). Baseline characteristics are listed in Supplementary Table S3. The median age of the cohort was 59.0 (IQR 48.0–68.0). VSRR patients were younger with fewer comorbidities than CVG patients (all *P* < 0.05). AAAD occurred in 20.2% with no significant difference between VSRR and CVG (*P* = 0.306), and TAR/PAR was performed at the similar frequency between both groups (*P* = 0.217). Compared to CVG, VSRR had a longer cardiopulmonary bypass (CPB) and aortic cross-clamp times (*P* < 0.001 for both) (Supplementary Table S4).

### Short- and long-term outcomes: all patients

**Figure 1:**
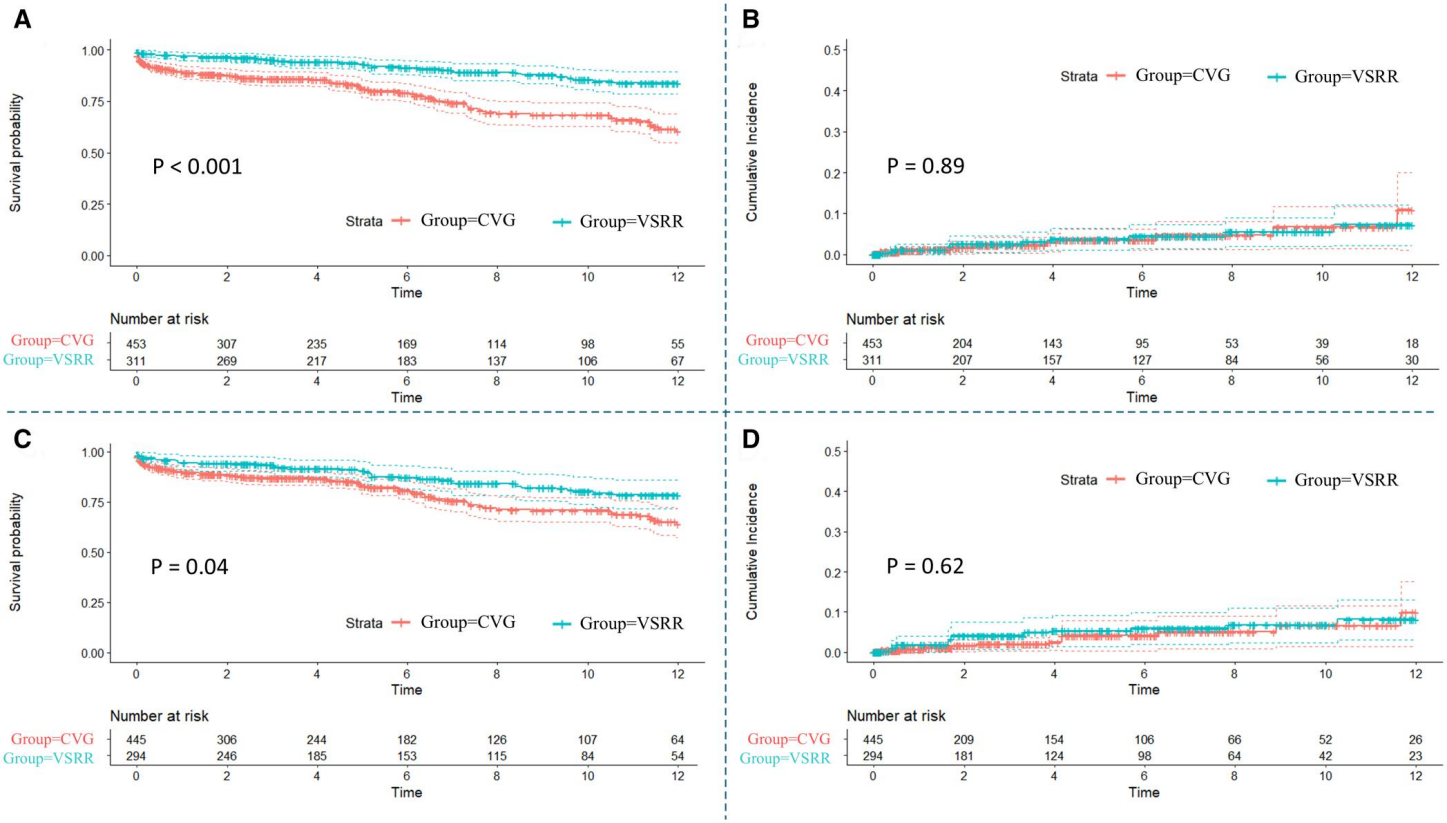
Kaplan–Meier analysis of survival (**A**, **C**) and the cumulative incidence of aortic valve reintervention (**B**, **D**) in patients after valve-sparing root replacement (VSRR) and aortic root replacement with composite valve graft (CVG). A and B, before IPTW; C and D, after IPTW

The VSRR cohort had a lower in-hospital mortality rate (*P* = 0.006) with increased uneventful recovery (*P* < 0.001) (Supplementary Table S5). The median duration of follow-up was 5.28 years for survival status (IQR, 2.0–10.5 years). VSRR was associated with higher long-term survival (*P* < 0.001) (Fig. 1A). Multivariate cox regression found that VSRR was associated with higher long-term survival (hazard ratio [HR] 0.505, VSRR compared to CVG, confidence interval [CI] 0.348–0.734, *P* < 0.001) (Table 1). The E-value for this finding was 3.37, suggesting that the finding is robust regarding potential confounding from unmeasured variables. The cumulative incidence of aortic valve reintervention was not significant difference after VSRR and CVG (*P* = 0.89) (Fig. 1B). A total of 24 cases (VSRR: 11, CVG: 13) required reintervention (Supplementary Table S6). The reasons for reintervention were as follows: AI in 7 patients (VSRR: 4, CVG: 3), aortic stenosis in 6 patients (VSRR: 2, CVG: 4), infection in 10 patients (VSRR: 5, CVG: 5) and pseudoaneurysm in 1 patient (CVG: 1).

**Table 1:** Multivariable analysis of factors associated with long-term mortality

	Hazard ratio (95% CI)	*P*-value
Age	1.027 (1.022–1.031)	<0.001
Female	1.532 (1.383–1.697)	<0.001
Diabetes	1.402 (1.197–1.642)	<0.001
CKD	1.700 (1.134–2.548)	0.010
Hypertension	1.018 (0.833–1.244)	0.864
Peripheral vascular disease	1.677 (1.022–2.752)	0.041
LVEF	0.971 (0.966–0.977)	<0.001
BAV	0.861 (0.800–0.923)	<0.001
AAAD	1.560 (1.188–2.049)	0.001
Elective	0.943 (0.660–1.349)	0.749
CABG	1.127 (0.983–1.291)	0.086
Mitral valve surgery	1.394 (1.323–1.469)	<0.001
Antegrade cerebral perfusion	0.500 (0.373–0.671)	<0.001
CPB time	1.004 (1.001–1.008)	0.007
VSRR	0.505 (0.348–0.734)	<0.001

CABG: coronary artery bypass; CKD: chronic kidney disease; LVEF: left ventricular ejection fraction.

**Table 2:** Baseline characteristics in adjusted cohort

	After IPTW (*n* = 738.2)			
	VSRR (*n* = 293.6)	CVG (*n* = 444.6)	*P*-value	SMD
Age, years	57.0 [47.0, 66.0]	59.0 [47.0, 68.0]	0.203	0.088
Female	48.7 (16.6)	74.0 (16.6)	0.984	0.002
BMI, kg/m^2^	28.1 [25.2, 32.0]	28.1 [24.9, 32.4]	0.599	0.006
Diabetes	29.6 (10.1)	49.0 (11.0)	0.733	0.030
Dyslipidaemia	146.6 (49.9)	223.4 (50.2)	0.937	0.007
CKD	54.2 (18.5)	87.3 (19.6)	0.738	0.029
Dialysis	2.2 (0.8)	4.7 (1.0)	0.726	0.030
Hypertension	226.8 (77.2)	346.1 (77.8)	0.863	0.014
Cerebrovascular disease	21.2 (7.2)	36.0 (8.1)	0.703	0.033
Peripheral arterial disease	17.1 (5.8)	29.3 (6.6)	0.713	0.032
Connective tissue disorder	9.2 (3.1)	12.4 (2.8)	0.815	0.019
LVEF	55.0 [54.0, 60.0]	55.0 [50.0, 60.0]	0.805	0.075
AI > Moderate	192.7 (65.6)	306.0 (68.7)	0.397	0.068
BAV	80.3 (27.4)	128.9 (29.0)	0.657	0.037
AAAD	62.4 (21.3)	92.8 (20.9)	0.915	0.009
Elective	152.3 (51.9)	227.8 (51.2)	0.880	0.013
Surgical year			0.665	0.075
2004–2009	94.3 (32.1)	127.8 (28.7)		
2010–2015	111.7 (38.0)	174.5 (39.3)		
2016–2021	87.7 (29.9)	142.3 (32.0)		

BMI: body mass index; CKD: chronic kidney disease; CVG: composite valve graft root replacement; LVEF: left ventricular ejection fraction.

**Table 3:** Operative characteristics in adjusted cohort

	After IPTW (*n* = 738.2)			
	VSRR (*n* = 293.6)	CVG (*n* = 444.6)	*P*-value	SMD
Concomitant cardiac surgery				
CABG	48.1 (16.4)	83.6 (18.8)	0.471	0.064
Mitral valve surgery	4.8 (1.6)	12.8 (2.9)	0.229	0.084
Aortic arch surgery				
Hemiarch	272.7 (82.9)	418.6 (94.1)	0.521	0.052
TAR/PAR	21.0 (7.1)	26.0 (5.9)	0.521	0.052
PAR	14.8 (5.1)	12.8 (2.9)	0.167	
TAR-FET	0.9 (0.3)	0.8 (0.2)	0.703	
Aortic valve replacement				
Mechanical	0 (0.0)	78.0 (17.6)	< 0.001	
Bioprosthetic	0 (0.0)	366.5 (82.4)	< 0.001	
Cerebral perfusion			0.221	
Only DHCA	30.4 (10.4)	30.0 (6.7)		
Antegrade	236.9 (80.7)	370.9 (83.4)		
Only retrograde	25.2 (8.6)	43.7 (9.8)		
Cardiopulmonary bypass				
CPB time, min	216.4 [163.1, 250.7]	193.8 [156.0, 236.0]	0.006	
Cross-clamp time, min	191.3 [132.0, 220.7]	161.0 [132.6, 192.4]	< 0.001	
Cerebral perfusion time, min	19.0 [12.5,24.0]	21.0 [15.2,27.0]	0.003	
Lowest temperature, degrees	27.1 [24.1,28.0]	26.2 [22.0,28.0]	0.017	

CABG: coronary artery bypass grafting; CVG: composite valve graft root replacement; DHCA: deep hypothermic cardiac arrest; FET: frozen elephant trunk; PAR: partial arch replacement; TAR: total arch replacement.

**Table 4:** Short-term outcomes in adjusted cohort

	After IPTW (*n* = 738.2)		
	VSRR (*n* = 293.6)	CVG (*n* = 444.6)	*P*-value
In-hospital mortality	7.3 (2.5)	21.9 (4.9)	0.195
Uneventful recovery	232.1 (79.0)	299.0 (67.3)	0.002
Reoperation for bleeding	13.6 (4.6)	46.9 (10.6)	0.005
Stroke	12.0 (4.1)	27.9 (6.3)	0.347
Prolonged ventilation	45.6 (15.5)	114.8 (25.8)	0.005
Renal failure	12.9 (4.4)	38.8 (8.7)	0.040
PPM implantation	3.6 (1.2)	6.5 (1.5)	0.785

CVG: composite valve graft root replacement; PPM: permanent pacemaker.

### Outcomes in IPTW-adjusted groups

IPTW created well-balanced groups with SMD < 0.10 for baseline and operative characteristics (Tables 2 and 3). In adjusted groups with IPTW, VSRR still had longer CPB and cross-clamp times (*P* = 0.006, and *P* < 0.001, respectively). In-hospital mortality was similar in the VSRR and CVG (VSRR: 2.5%, CVG: 4.9%, *P* = 0.195), whereas uneventful recovery was higher after VSRR (79.0% vs 67.3%, *P* = 0.002), exemplified by lower rates of reoperation for bleeding (4.6% vs 10.6%, *P* = 0.005), renal failure (4.4% vs 8.7%, *P* = 0.040) and prolonged ventilation (15.5% vs 25.8%, *P* = 0.005) (Table 4). Long-term survival was still higher after VSRR (*P* = 0.04) (Fig. 1C). At 12-year, the survival rate was 78.5% [71.7–86.1%] after VSRR, and 64.2% [57.4–71.6%] after CVG. However, the incidence of aortic valve reintervention was similar (*P* = 0.62): at 12-year, the rate was 8.2% [3.0–13.1%] for VSRR and 9.9% [1.4–17.7%] for CVG (Fig. 1D).

### Subgroups analysis in patients with uneventful recovery

**Figure 2:**
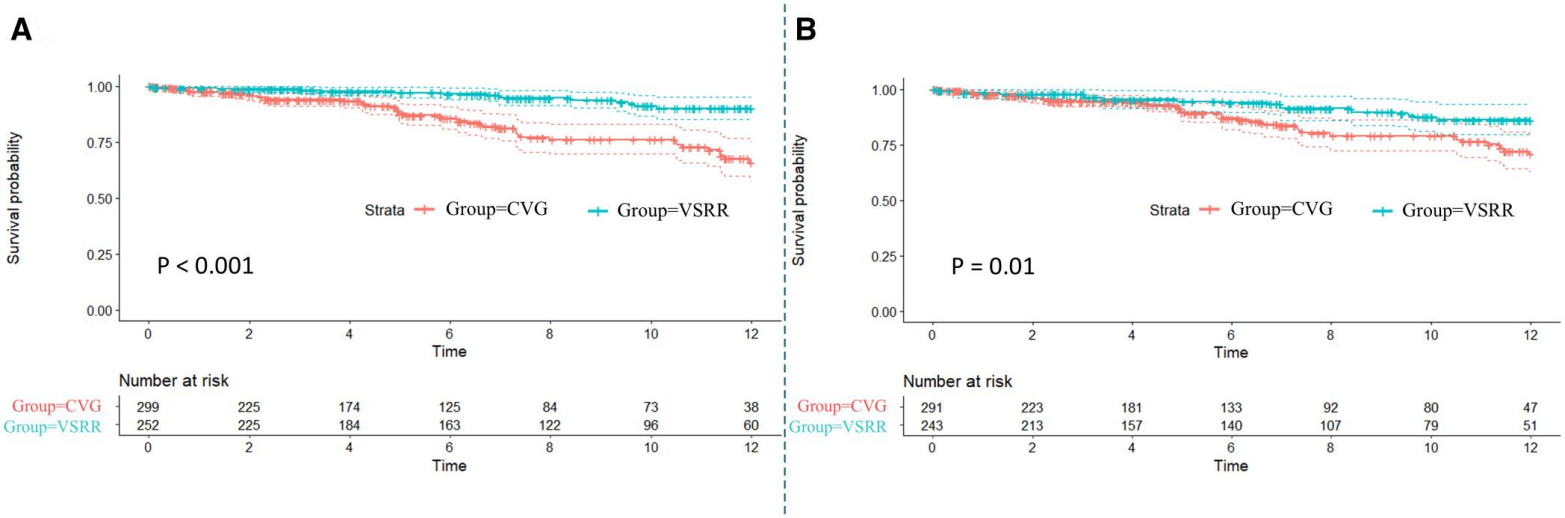
Sensitive analysis for long-term mortality. (**A**, **B**) Kaplan–Meier analysis of survival after valve-sparing root replacement (VSRR) and aortic root replacement with composite valve graft (CVG) in patients with uneventful recovery. A, before IPTW; B, after IPTW

A total of 551 patients were included in this subgroup analysis. IPTW created well-balanced groups for baseline and operative characteristics (Supplementary Tables S7 and S8). In IPTW-adjusted groups, long-term survival was still higher after VSRR (*P* = 0.01) (Fig. 2B). At 12-year, the survival rate was 86.4% [80.1–93.5%] after VSRR, and 71.7% [63.1–79.8%] after CVG.

### Subgroups analysis in patients with concomitant ARR and hemiarch replacement

A total of 714 patients underwent concomitant ARR and hemiarch replacement (VSRR: *n* = 286, CVG: *n* = 428). IPTW yielded well-balanced groups (Supplementary Tables S9 and S10). After IPTW, in-hospital mortality was similar in the VSRR and CVG (VSRR: 2.6%, CVG: 4.9%, *P* = 0.268) (Supplementary Table S11). Furthermore, in IPTW-adjusted groups, long-term survival was significant difference between VSRR and CVG (*P* = 0.027): at 10-year, the rate was 79.3% [72.2–87.0%] for VSRR and 71.3% [65.4–77.6%] for CVG (Supplementary Fig. S3).

## DISCUSSION

Our data suggest that, in comparison to CVG, VSRR was associated with acceptable short- and long-term outcomes in concomitant aortic root and arch surgery. This study is the first to examine the influence of sparing the aortic valve on short- and long-term outcomes, extending beyond 10 years, during concomitant ARR and arch surgery with a large cohort size. Several studies have reported outcomes on concomitant ARR and arch surgery, however, literature examining the influence of sparing the aortic valve on outcome during such complex procedures is limited [22–29]. Li *et al.* [29] found that in 148 patients with concomitant root and TAR at the time of AAAD repair, the overall survival rate (VSRR: 82.9% vs CVG: 89.2%, *P* = 0.63) and freedom from valve-related events (VSRR: 96.3% vs CVG: 94.5%, *P* = 0.34) were similar at 5 years; however, long-term outcomes—particularly those extending beyond 10 years—remain scarce. Therefore, we believe that the present multicentre study provides important short- and long-term data supporting the increasing use of VSRR for patients who require concomitant ARR and arch replacement.

Our data showed favourable short-term outcomes after VSRR. The in-hospital mortality of the entire cohort was 4.2%, with VSRR at 1.6%, which were comparable to previous studies reporting rates between 2.6% and 9% for concomitant aortic root and arch surgery [22–29]. In adjusted groups, in-hospital mortality was comparable, and the rate of uneventful recovery was higher after VSRR. This finding might appear controversial as the VSRR cohort had longer CPB and aortic cross-clamp time, both well-known independent risk factors of postoperative morbidity and mortality [30, 31]. Previous studies reported similar early mortality and morbidity in VSRR and CVG [6–10]. Meta-analysis showed a nonsignificant trend towards reduced early mortality and complications with VSRR [11]. Overall, we believe that unmeasurable confounders, such as differences in patient selection based on a ‘surgeon’s eyeball test’ and insufficient statistical power, may account for these differences.

The present study also demonstrated that VSRR was not associated with worse long-term outcome than CVG. E-values from the Cox regression showed that major confounders would be required to negate our statistical results. Our sensitivity analysis of patients with uneventful recovery further supported the robustness of our findings in comparing long-term survival after removing patients with complicated postoperative courses. Long-term aortic valve reintervention rates were similar in both groups. Several studies have reported increased long-term cardiac mortality and morbidity with CVG when compared to VSRR, attributed to complications related to aortic valve dysfunction [5–9]. Conversely, Elbatarny *et al.* [11] found that VSRR was associated with lower long-term mortality and equivalent durability compared with CVG in a large meta-analysis, although not without acknowledging the presence of selection bias. Even in the setting of this selection bias and other unmeasured confounders, we believe our data show that sparing the aortic valve can be a safe option for appropriately selected patients undergoing concomitant ARR and arch surgery.

### Limitation

This study has several limitations. First, this is a retrospective study at two large-volume aortic centres, limiting generalization of the findings. Second, even with our large numbers of patients of concomitant aortic root and arch surgery, the event rate for reinterventions was small, limiting the statistical power of our analysis. Third, because postoperative medical management, particularly echocardiography data, were not collected uniformly and thus are not included in this study, it remains unknown how many patients had significant valvular dysfunction with or without reintervention. Although we followed more than 26.4% of patients for over 10 years postoperatively, longer follow-up is needed to better ascertain the lifelong differences between these procedures given the durability of bioprosthetic aortic valves. Fourth, the entire cohort consists of patients with no absolute contraindications for VSRR. However, since surgeons and centres have different thresholds for performing VSRR in cases of root aneurysm, patients with similar characteristics and aortic valves were managed differently. As a result, we were unable to establish a universal indication for VSRR in this study. Furthermore, although we matched several factors, including baseline characteristics, surgical procedure and concomitant surgery, the heterogeneity could lead to varying intraoperative outcomes across the groups, which may impact our study findings. Fifth, with a lack of data on the cause of deaths, we could not elucidate the factors behind better early- and long-term survival after VSRR. Although our high E-value suggests the robustness of the observed associations, our adjusted comparisons are subject to the influence of unmeasured confounders, such as decision-making processes among surgeons, which are based on their experience.

## CONCLUSION

VSRR was associated with acceptable short- and long-term outcomes with concomitant aortic arch surgery. In appropriately selected patients, a valve-sparing operation appears to be a safe option for those who require concomitant ARR and arch replacement.

## Supplementary Material

ivaf045_Supplementary_Data

## Data Availability

The data underlying this article will be shared on reasonable request to the corresponding author.

## ABBREVIATIONS

AAAD: Acute type A aortic dissection
ARR: Aortic root replacement
CPB: Cardiopulmonary bypass
CVG: Composite valve graft root replacement
IPTW: Inverse probability of treatment weighting
PAR: Partial arch replacement
PS: Propensity score
SMD: Standardized mean differences
STS: Society of Thoracic Surgeons
TAR: Total arch replacement
VSRR: Valve-sparing aortic root replacement
