# Supplementary material for: Assessment of short- and long-term outcomes of aortic valve-sparing operation at concomitant aortic root and arch repair
Source: Interdiscip Cardiovasc Thorac Surg. 2025 Feb 26;40(7):ivaf045. doi: 10.1093/icvts/ivaf045 (PMC12245402; doi:10.1093/icvts/ivaf045)
Supplement: ivaf045_Supplementary_Data [file ivaf045_supplementary_data.zip › Supplemental Tables 0115.docx]

Supplemental Table 1. Missing variables

| Variables | Missing, n (%) |
| --- | --- |
| Body mass index | 3 (0.4) |
| Dyslipidemia | 22 (2.9) |
| Chronic kidney disease | 5 (0.7) |
| Cerebrovascular disease | 2 (0.3) |
| Peripheral artery disease | 2 (0.3) |
| Aortic insufficiency | 8 (1.0) |
| Bicuspid aortic valve | 1 (0.1) |
| Left ventricular ejection fraction | 45 (5.9) |
| Cerebral perfusion strategy | 12 (1.6) |
| Cerebral perfusion time | 56 (7.3) |
| Cross-clamp time | 1 (0.1) |
| Lowest temperature | 10 (1.3) |

Supplemental Table 2. Univariable analysis of factors associated with long-term mortality.

|  | **Hazard Ratio (95% CI)** | **p-value** |
| --- | --- | --- |
| Age | 1.033 (1.020-1.047) | < 0.001 |
| Female | 1.467 (0.988-2.123) | 0.057 |
| BMI | 0.983 (0.953-1.008) | 0.226 |
| Diabetes | 2.691 (1.756-3.990) | < 0.001 |
| Dyslipidemia | 0.921 (0.661-1.278) | 0.624 |
| CKD | 2.351 (1.638-3.323) | <0.001 |
| Hypertension | 1.855 (1.211-2.972) | 0.004 |
| Cerebrovascular disease | 1.211 (0.635-2.093) | 0.537 |
| Peripheral vascular disease | 2.434 (1.362-4.040) | 0.004 |
| Connective tissue disorder | 1.559 (0.552-3.245) | 0.362 |
| Left ventricular ejection fraction | 0.968 (0.955-0.983) | < 0.001 |
| AI>Moderate | 1.286 (0.906-1.859) | 0.162 |
| BAV | 0.701 (0.468-1.022) | 0.065 |
| AAAD | 2.395 (1.695-3.347) | <0.001 |
| Elective | 0.538 (0.383-0.749) | <0.001 |
| Surgical year (Ref. 2004-2009) |  |  |
| 2010-2015 | 0.743 (0.506-1.842) | 0.124 |
| 2016-2021 | 0.932 (0.555-1.529) | 0.782 |
| CABG | 2.028 (1.398-2.887) | < 0.001 |
| Mitral valve surgery | 2.221 (1.045-4.111) | 0.038 |
| TAR/PAR | 1.034 (0.488-1.915) | 0.923 |
| Antegrade cerebral perfusion | 0.690 (0.459-1.078) | 0.100 |
| CPB time | 1.005 (1.003-1.007) | < 0.001 |
| VSRR | 0.344 (0.232-0.498) | < 0.001 |

AAAD; acute type A aortic dissection, AI; aortic insufficiency; BAV; bicuspid aortic valve, BMI, body mass index; CABG; coronary artery bypass, CI; confidence interval; CKD, chronic kidney disease; CPB; cardiopulmonary bypass time, LVEF; left ventricular ejection fraction; PAR, partial arch replacement; TAR, total arch replacement; VSRR, valve sparing aortic root replacement

Supplemental Table 3. List of patients requiring reintervention

| Patient  Number | Index surgery | Duration from prior surgery (Year) | Procedure | Indication for reintervention |
| --- | --- | --- | --- | --- |
| 1 | VSRR | 0.18 | CVG | Infection |
| 2 | VSRR | 0.39 | CVG | Infection |
| 3 | VSRR | 0.50 | AVR | AI |
| 4 | VSRR | 1.65 | CVG | Infection |
| 5 | VSRR | 1.70 | CVG | Infection |
| 6 | VSRR | 1.72 | CVG | Infection |
| 7 | VSRR | 3.32 | AVR | AI |
| 8 | VSRR | 3.96 | AVR | AI |
| 9 | VSRR | 5.71 | AVR | AI |
| 10 | VSRR | 7.86 | AVR | AS |
| 11 | VSRR | 10.27 | AVR | AS |
| 12 | CVG with bioprosthetic aortic valve | 0.03 | CVG | Infection |
| 13 | CVG with bioprosthetic aortic valve | 0.16 | CVG | Infection |
| 14 | CVG with bioprosthetic aortic valve | 1.12 | CVG | Infection |
| 15 | CVG with bioprosthetic aortic valve | 3.89 | AVR | AI |
| 16 | CVG with bioprosthetic aortic valve | 4.16 | AVR | AS |
| 17 | CVG with bioprosthetic aortic valve | 6.29 | AVR | AS |
| 18 | CVG with bioprosthetic aortic valve | 68.92 | AVR | AI |
| 19 | CVG with bioprosthetic aortic valve | 11.68 | TAVR | AS |
| 20 | CVG with bioprosthetic aortic valve | 12.39 | TAVR | AS |
| 21 | CVG with bioprosthetic aortic valve | 12.41 | AVR | AI |
| 22 | CVG with mechanical aortic valve | 0.62 | CVG | Infection |
| 23 | CVG with mechanical aortic valve | 1.70 | CVG | Formation of pseudoaneurysm |
| 24 | CVG with mechanical aortic valve | 2.32 | AVR | Infection |

AI, aortic insufficiency; AVR, aortic valve replacement; CVG, composite valve graft aortic replacement; VSRR, valve sparing aortic root replacement.

Supplemental Table 4. Baseline characteristics in patients with uneventful recovery

|  | All patients (n=551) | |  | After IPTW (n=533.6) | |  |  |
| --- | --- | --- | --- | --- | --- | --- | --- |
|  | VSRR (n=252) | CVG (n=299) | P-value | VSRR (n=243.2) | CVG (n=290.4) | p-value | SMD |
| Age, years | 53.0 [44.8,63.0] | 62.0 [52.0,70.5] | < 0.001 | 57.0 [47.0,65.0] | 59.0 [47.0,68.0] | 0.310 | 0.066 |
| Female | 35 (13.9) | 53 (17.7) | 0.268 | 34.7 (14.9) | 43.3 (14.9) | 0.844 | 0.018 |
| BMI, kg/m^2^ | 28.1 [25.4,31.6] | 27.8 [24.5,31.4] | 0.140 | 28.1 [25.2,31.6] | 27.8 [24.5,31.7] | 0.287 | 0.008 |
| Diabetes | 16 (6.3) | 37 (12.4) | 0.025 | 19.7 (8.1) | 27.1 (9.3) | 0.647 | 0.044 |
| Dyslipidemia | 118 (46.8) | 169 (56.5) | 0.029 | 124.3 (51.1) | 151.1 (52.0) | 0.850 | 0.018 |
| CKD | 23 (9.1) | 50 (16.7) | 0.013 | 32.6 (13.4) | 39.5 (13.6) | 0.954 | 0.006 |
| Hypertension | 182 (72.2) | 239 (79.9) | 0.043 | 182.7 (75.1) | 218.6 (75.3) | 0.972 | 0.003 |
| Cerebrovascular disease | 11 (4.4) | 33 (11.0) | 0.007 | 13.6 (5.6) | 23.5 (8.1) | 0.304 | 0.100 |
| Peripheral arterial disease | 8 (3.2) | 19 (6.4) | 0.127 | 10.3 (4.2) | 14.7 (5.1) | 0.698 | 0.039 |
| Connective tissue disorder | 6 (2.4) | 8 (2.7) | 1.000 | 7.7 (3.2) | 7.9 (2.7) | 0.810 | 0.025 |
| LVEF | 55.0 [54.8,60.0] | 55.0 [50.0,60.0] | 0.022 | 55.0 [54.0,60.0] | 55.0 [51.3,60.0] | 0.983 | 0.043 |
| AI >Moderate | 127 (50.4) | 231 (77.3) | < 0.001 | 158.6 (65.2) | 202.7 (69.8) | 0.304 | 0.098 |
| BAV | 76 (30.2) | 103 (34.4) | 0.327 | 76.3 (31.4) | 96.6 (33.3) | 0.678 | 0.040 |
| AAAD | 30 (11.9) | 40 (13.4) | 0.697 | 30.0 (12.4) | 36.1 (12.4) | 0.979 | 0.002 |
| Elective | 151 (59.9) | 169 (56.5) | 0.472 | 144.9 (59.6) | 172.7 (59.5) | 0.979 | 0.003 |
| Surgical year |  |  | < 0.001 |  |  | 0.804 | 0.064 |
| 2004-2009 | 86 (34.1) | 68 (22.7) |  | 71.6 (29.4) | 77.4 (26.7) |  |  |
| 2010-2015 | 106 (42.1) | 118 (39.5) |  | 98.0 (40.3) | 119.5 (41.2) |  |  |
| 2016-2021 | 60 (23.8) | 113 (37.8) |  | 736.6 (30.3) | 93.4 (32.2) |  |  |

AAAD; acute type A aortic dissection, AI; aortic insufficiency, BAV; bicuspid aortic valve, BMI; body mass index, CKD, chronic kidney disease, CVG, composite valve graft aortic replacement; IPTW, inverse probability of treatment weighting; LVEF, left ventricular ejection fraction; SMD, standardized mean difference; VSRR, valve sparing aortic root replacement.

Supplemental Table 5. Operative characteristics in patients with uneventful recovery

|  | All patients (n=551) | | | After IPTW (n=533.6) | | |  |
| --- | --- | --- | --- | --- | --- | --- | --- |
|  | VSRR (n=252) | CVG (n=299) | P-value | VSRR (n=243.2) | CVG (n=290.4) | p-value | SMD |
| Concomitant cardiac surgery | | | | | | |  |
| CABG | 25 (9.9) | 50 (16.7) | 0.028 | 32.0 (13.2) | 41.5 (14.3) | 0.737 | 0.033 |
| Mitral valve surgery | 4 (1.6) | 9 (3.0) | 0.415 | 4.4 (1.8) | 6.4 (2.2) | 0.768 | 0.029 |
| Aortic arch surgery | | | | | | | |
| Hemiarch | 238 (94.4) | 285 (95.3) | 0.787 | 230.9 (95.0) | 277.4 (95.5) | 0.769 | 0.027 |
| TAR/PAR | 14 (5.6) | 14 (4.7) | 0.787 | 12.2 (5.0) | 13.0 (4.5) | 0.769 | 0.027 |
| PAR | 10 (4.0) | 7 (2.3) | 0.394 | 9.2 (3.8) | 6.1 (2.1) | 0.269 | 0.099 |
| Aortic valve replacement | | | | | | | |
| Mechanical | 0 (0.0) | 36 (12.0) | < 0.001 | 0 (0.0) | 46.4 (16.0) | < 0.001 |  |
| Bioprosthetic | 0 (0.0) | 263 (88.0) | < 0.001 | 0 (0.0) | 244.0 (84.0) | < 0.001 |  |
| Cerebral Perfusion | | |  |  |  |  |  |
| Only DHCA | 19 (7.5) | 10 (3.3) | 0.045 | 16.4 (6.7) | 11.0 (3.8) | 0.162 |  |
| Antegrade | 208 (82.5) | 255 (85.3) | 0.448 | 200.6 (82.4) | 245.4 (84.4) | 0.575 |  |
| Only Retrograde | 25 (9.9) | 34 (11.4) | 0.682 | 26.4 (10.8) | 34.1 (11.7) | 0.774 |  |
| Cardiopulmonary bypass | | | | | | | |
| CPB time, minutes | 211.0 [164.8,244.3] | 183.0  [151.0, 210.0] | < 0.001 | 212.0 [161.0,235.0] | 183.0 [151.0,212.0] | < 0.001 |  |
| Cross-clamp time,  minutes | 189.5 [135.0,219.0] | 157.0 [127.0,183.0] | < 0.001 | 183.5 [133.4,216.0] | 157.0 [127.0,184.3] | < 0.001 |  |
| Cerebral perfusion time,  minutes | 19.00  [11.75, 23.00] | 21.00  [16.00, 25.00] | 0.004 | 18.86  [12.00, 23.00] | 20.00  [14.00, 24.56] | 0.029 |  |
| Lowest temperature, degrees | 28.0 [24.9,28.0] | 26.9 [24.0,28.0] | 0.009 | 28.0 [24.6,28.0] | 27.0 [24.0,28.0] | 0.025 |  |

CABG, coronary artery bypass grafting; CPB, cardiopulmonary bypass; CVG, composite valve graft aortic replacement; DHCA, deep hypothermic cardiac arrest; FET, frozen elephant trunk; IPTW, inverse probability of treatment weighting; SMD, standardized mean difference; PAR; partial arch replacement, TAR; total arch replacement; VSRR, valve sparing aortic root replacement.

**Supplemental Table 6**. Baseline characteristics in patients with concomitant aortic root replacement and hemiarch replacement

|  | All patients (n=714) | |  | After IPTW (n=689.3) | |  |  |
| --- | --- | --- | --- | --- | --- | --- | --- |
|  | VSRR (n=286) | CVG (n=428) | P-value | VSRR (n=267.6) | CVG (n=421.7) | p-value | SMD |
| Age, years | 53.0 [44.0,63.0] | 62.0 [52.0,71.0] | < 0.001 | 57.0 [47.0,66.0] | 59.0 [47.0,68.0] | 0.194 | 0.090 |
| Female | 39 (13.6) | 76 (17.8) | 0.173 | 39.1 (14.6) | 65.6 (15.6) | 0.761 | 0.026 |
| BMI, kg/m^2^ | 28.3 [25.4,32.3] | 28.2 [250,32.0] | 0.323 | 28.3 [25.2,32.0] | 28.2 [25.1,32.3] | 0.656 | 0.009 |
| Diabetes | 19 (6.6) | 60 (14.0) | 0.003 | 23.9 (8.9) | 45.9 (10.9) | 0.466 | 0.066 |
| Dyslipidemia | 134 (46.9) | 227 (53.0) | 0.123 | 132.2 (49.4) | 210.4 (49.9) | 0.910 | 0.010 |
| CKD | 33 (11.5) | 104 (24.3) | < 0.001 | 48.3 (18.0) | 82.5 (19.6) | 0.674 | 0.039 |
| Dialysis | 2 (0.7) | 4 (0.9) | 1.000 | 2.4 (0.9) | 4.2 (1.0) | 0.917 | 0.009 |
| Hypertension | 210 (73.4) | 345 (80.6) | 0.030 | 205.8 (76.9) | 326.2 (77.4) | 0.897 | 0.011 |
| Cerebrovascular disease | 15 (5.2) | 41 (9.6) | 0.049 | 20.1 (7.5) | 11.1 (2.6) | 0.763 | 0.028 |
| Peripheral arterial disease | 11 (3.8) | 29 (6.8) | 0.133 | 12.7 (4.8) | 23.4 (5.5) | 0.696 | 0.035 |
| Connective tissue disorder | 7 (2.4) | 11 (2.6) | 1.000 | 7.7 (2.9) | 11.1 (2.6) | 0.865 | 0.015 |
| LVEF | 55.0 [55.0,60.0] | 55.0 [50.0,60.0] | 0.002 | 55.0 [54.0,60.0] | 55.0 [50.0,60.0] | 0.764 | 0.069 |
| AI >Moderate | 148 (51.7) | 322 (75.2) | < 0.001 | 175.0 (65.4) | 289.5 (68.7) | 0.404 | 0.020 |
| BAV | 79 (27.6) | 133 (31.3) | 0.365 | 76.0 (28.4) | 124.7 (29.6) | 0.760 | 0.026 |
| AAAD | 42 (14.7) | 93 (21.7) | 0.024 | 52.7 (19.7) | 83.8 (19.9) | 0.954 | 0.005 |
| Elective | 163 (57.0) | 215 (50.2) | 0.090 | 142.9 (53.4) | 221.0 (52.4) | 0.820 | 0.020 |
| Surgical year |  |  | 0.001 |  |  | 0.621 | 0.084 |
| 2004-2009 | 103 (36.0) | 110 (25.7) |  | 88.8 (33.2) | 124.0 (29.4) |  |  |
| 2010-2015 | 113 (39.5) | 161 (37.6) |  | 101.6 (38.0) | 165.1 (39.2) |  |  |
| 2016-2021 | 70 (24.5) | 157 (36.7) |  | 77.2 (28.9) | 132.6 (31.4) |  |  |

AAAD; acute type A aortic dissection, AI; aortic insufficiency, BAV; bicuspid aortic valve, BMI; body mass index, CKD, chronic kidney disease, CVG, composite valve graft aortic replacement; IPTW, inverse probability of treatment weighting; LVEF, left ventricular ejection fraction; SMD, standardized mean difference; VSRR, valve sparing aortic root replacement.

Supplemental Table 7. Operative characteristics in patients with concomitant aortic root replacement and hemiarch replacement

|  | All patients (n=714) | | | After IPTW (n=689.3) | | |  |
| --- | --- | --- | --- | --- | --- | --- | --- |
|  | VSRR (n=286) | CVG (n=428) | P-value | VSRR (n=267.6) | CVG (n=421.7) | p-value | SMD |
| Concomitant cardiac surgery | | | | | | |  |
| CABG | 32 (11.2) | 100 (23.4) | < 0.001 | 45.4 (17.0) | 81.2 (19.2) | 0.523 | 0.059 |
| Mitral valve surgery | 5 (1.7) | 18 (4.2) | 0.108 | 4.1 (1.5) | 12.6 (3.0) | 0.192 | 0.097 |
| Aortic arch surgery | | | | | | | |
| Hemiarch | 286 (100.0) | 428 (100.0) | 1.000 | 267.6 (100.0) | 421.7 (100.0) | 1.000 |  |
| Aortic valve replacement | | | | | | | |
| Mechanical | 0 (0.0) | 59 (13.8) | < 0.001 | 0.0 (0.0) | 76.2 (18.1) | < 0.001 |  |
| Bioprosthetic | 0 (0.0) | 369 (86.2) | < 0.001 | 0.0 (0.0) | 345.5 (81.9) | < 0.001 |  |
| Cerebral Perfusion | | | 0.045 |  |  | 0.077 |  |
| Only DHCA | 31 (10.8) | 26 (6.1) |  | 32.1 (12.0) | 28.0 (6.6) |  |  |
| Antegrade | 233 (81.5) | 358 (83.6) |  | 214.1 (80.0) | 349.5 (82.9) |  |  |
| Only Retrograde | 22 (7.7) | 44 (10.3) |  | 21.4 (8.0) | 44.2 (10.5) |  |  |
| Cardiopulmonary bypass | | | | | | | |
| CPB time, minutes | 214.0 [163.3,248.8] | 190.5 [155.0,229.0] | 0.001 | 216.0 [161.0,249.0] | 190.0 [154.0,229.0] | 0.003 |  |
| Cross-clamp time,  minutes | 191.0 [135.0,221.0] | 161.0 [131.5,189.0] | < 0.001 | 188.0 [133.4,219.2] | 160.0 [128.2,189.0] | < 0.001 |  |
| Cerebral perfusion time,  minutes | 19.0 [11.0,23.0] | 19.0 [15.0,25.0] | 0.001 | 17.8±14.3 | 20.2±12.7 | 0.030 |  |
| Lowest temperature,  degrees | 28.0 [24.8,28.0] | 26.6 [22.8,28.0] | 0.002 | 27.3 [24.1,28.0] | 26.6 [23.0,28.0] | 0.025 |  |

CABG, coronary artery bypass grafting; CPB, cardiopulmonary bypass; CVG, composite valve graft aortic replacement; DHCA, deep hypothermic cardiac arrest; FET, frozen elephant trunk; IPTW, inverse probability of treatment weighting; SMD, standardized mean difference; PAR; partial arch replacement, TAR; total arch replacement; VSRR, valve sparing aortic root replacement.

Table 8. Short-term outcomes in patients with concomitant aortic root replacement and hemiarch replacement

|  | All patients (n=714) | | | After IPTW (n=689.3) | | |
| --- | --- | --- | --- | --- | --- | --- |
|  | VSRR (n=286) | CVG (n=428) | P-value | VSRR (n=267.6) | CVG (n=421.7) | P-value |
| In-hospital Mortality | 4 (1.4) | 26 (6.1) | 0.004 | 7.0 (2.6) | 20.7 (4.9) | 0.268 |
| Uneventful Recovery | 238 (83.2) | 285 (66.6) | < 0.001 | 216.1 (80.8) | 287.2 (68.1) | 0.002 |
| Reoperation for bleeding | 14 (4.9) | 47 (11.0) | 0.007 | 11.9 (4.4) | 42.9 (10.2) | 0.007 |
| Stroke | 6 (2.1) | 24 (5.6) | 0.036 | 10.3 (3.8) | 26.5 (6.3) | 0.306 |
| Prolonged ventilation | 32 (11.2) | 113 (26.4) | < 0.001 | 35.6 (13.3) | 105.2 (25.0) | 0.002 |
| Renal failure | 12 (4.2) | 41 (9.6) | 0.011 | 12.1 (4.5) | 35.5 (8.4) | 0.082 |
| PPM implantation | 4 (1.4) | 8 (1.9) | 0.855 | 3.5 (1.3) | 6.6 (1.6) | 0.771 |

CVG, composite valve graft aortic replacement; IPTW, inverse probability of treatment weighting; PPM, permanent pacemaker; SMD, standardized mean difference; VSRR, valve sparing aortic root replacement.
