# Supplementary figures and images for: Assessment of short- and long-term outcomes of aortic valve-sparing operation at concomitant aortic root and arch repair
Source: Interdiscip Cardiovasc Thorac Surg. 2025 Feb 26;40(7):ivaf045. doi: 10.1093/icvts/ivaf045 (PMC12245402; doi:10.1093/icvts/ivaf045)

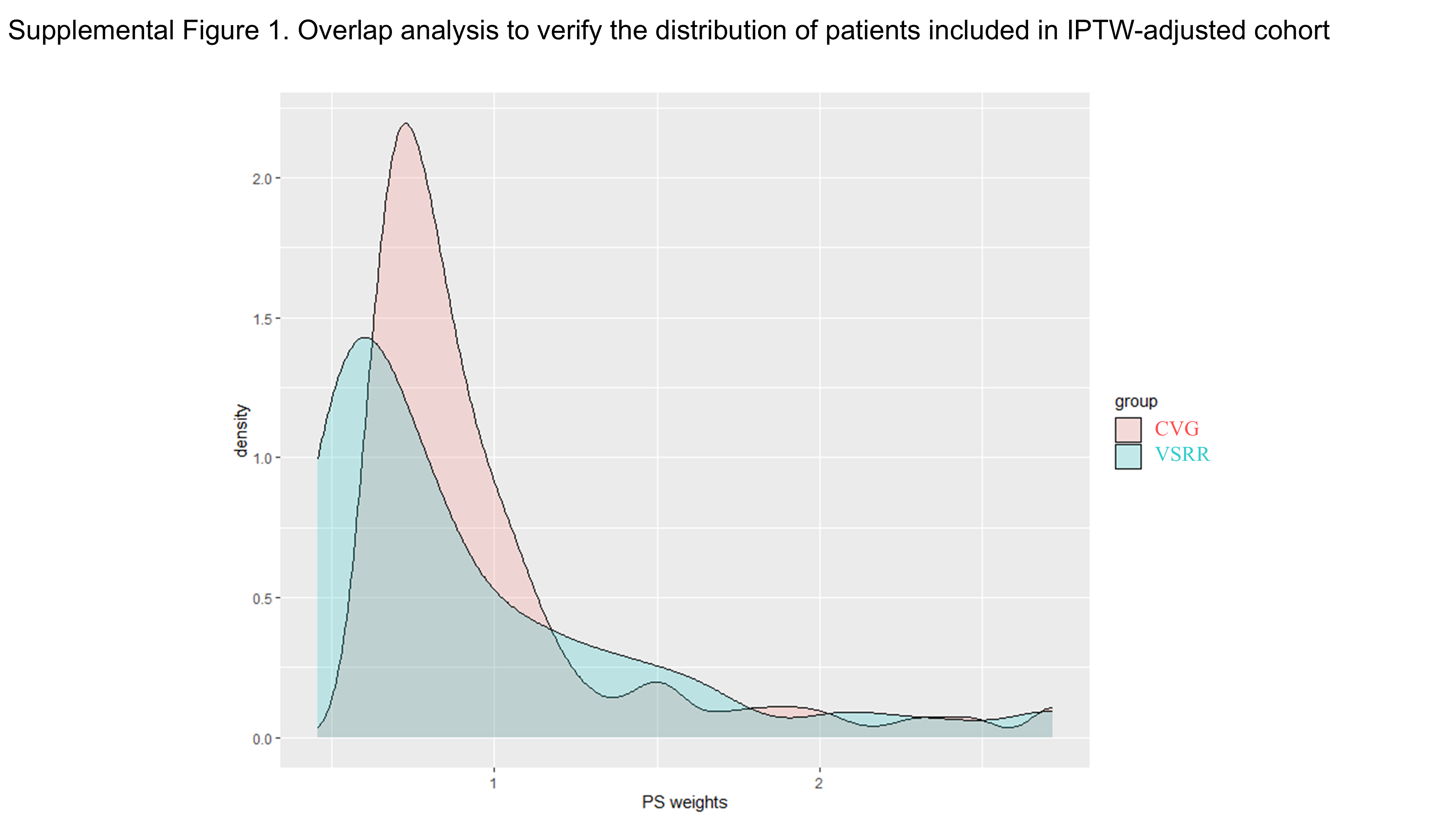

Supplement: ivaf045_Supplementary_Data [file ivaf045_supplementary_data.zip › Supplemental Figure 1.tif]

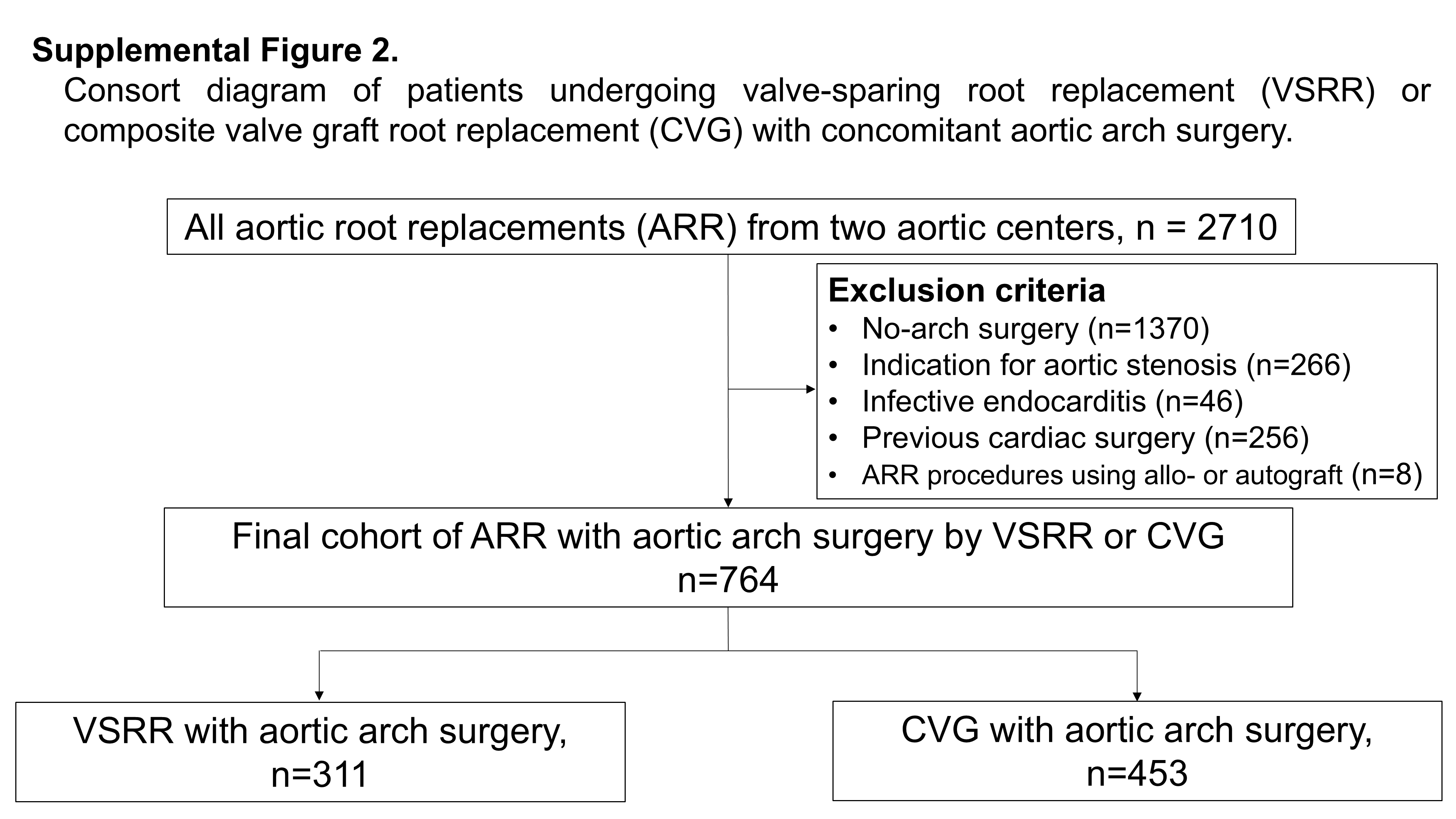

Supplement: ivaf045_Supplementary_Data [file ivaf045_supplementary_data.zip › Supplemental Figure 2.tif]

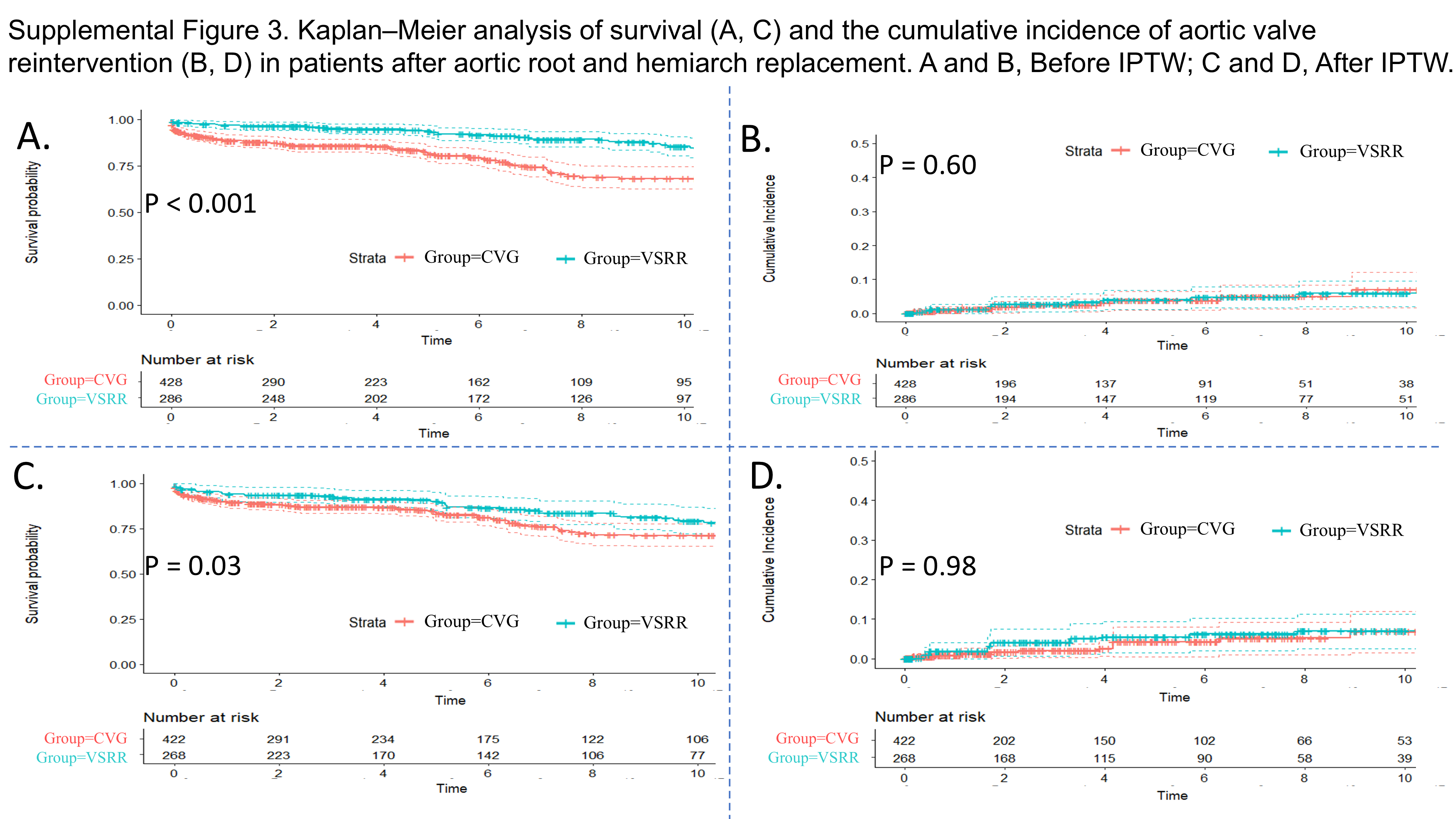

Supplement: ivaf045_Supplementary_Data [file ivaf045_supplementary_data.zip › Supplemental Figure 3.tif]
